# New onset delirium prediction using machine learning and long short-term memory (LSTM) in electronic health record

**DOI:** 10.1093/jamia/ocac210

**Published:** 2022-10-27

**Authors:** Siru Liu, Joseph J Schlesinger, Allison B McCoy, Thomas J Reese, Bryan Steitz, Elise Russo, Brian Koh, Adam Wright

**Affiliations:** Department of Biomedical Informatics, Vanderbilt University Medical Center, Nashville, Tennessee, USA; Division of Critical Care Medicine, Department of Anesthesiology, Vanderbilt University Medical Center, Nashville, Tennessee, USA; Department of Biomedical Informatics, Vanderbilt University Medical Center, Nashville, Tennessee, USA; Department of Biomedical Informatics, Vanderbilt University Medical Center, Nashville, Tennessee, USA; Department of Biomedical Informatics, Vanderbilt University Medical Center, Nashville, Tennessee, USA; Department of Biomedical Informatics, Vanderbilt University Medical Center, Nashville, Tennessee, USA; Department of Biomedical Informatics, Vanderbilt University Medical Center, Nashville, Tennessee, USA; Department of Biomedical Informatics, Vanderbilt University Medical Center, Nashville, Tennessee, USA

**Keywords:** deep learning, explainable machine learning, delirium, predictive models

## Abstract

**Objective:**

To develop and test an accurate deep learning model for predicting new onset delirium in hospitalized adult patients.

**Methods:**

Using electronic health record (EHR) data extracted from a large academic medical center, we developed a model combining long short-term memory (LSTM) and machine learning to predict new onset delirium and compared its performance with machine-learning-only models (logistic regression, random forest, support vector machine, neural network, and LightGBM). The labels of models were confusion assessment method (CAM) assessments. We evaluated models on a hold-out dataset. We calculated Shapley additive explanations (SHAP) measures to gauge the feature impact on the model.

**Results:**

A total of 331 489 CAM assessments with 896 features from 34 035 patients were included. The LightGBM model achieved the best performance (AUC 0.927 [0.924, 0.929] and *F*1 0.626 [0.618, 0.634]) among the machine learning models. When combined with the LSTM model, the final model’s performance improved significantly (*P* = .001) with AUC 0.952 [0.950, 0.955] and *F*1 0.759 [0.755, 0.765]. The precision value of the combined model improved from 0.497 to 0.751 with a fixed recall of 0.8. Using the mean absolute SHAP values, we identified the top 20 features, including age, heart rate, Richmond Agitation-Sedation Scale score, Morse fall risk score, pulse, respiratory rate, and level of care.

**Conclusion:**

Leveraging LSTM to capture temporal trends and combining it with the LightGBM model can significantly improve the prediction of new onset delirium, providing an algorithmic basis for the subsequent development of clinical decision support tools for proactive delirium interventions.

## INTRODUCTION

Delirium is an acute decline in cognitive function leading to confusion, which occurs in 29% to 65% of hospitalized older patients.^1–3^ Patients with delirium experience a serious constellation of neuropsychiatric symptoms, resulting in higher mortality, in-hospital falls, and the need for long-term care.^4–7^ The risk of mortality increases by 11% for every additional 48 h after the onset of delirium.^8^ In addition, delirium is associated with continued deterioration in cognitive function,^9^ as well as reduced functional status,^6^ and it negatively affects mental health status (eg, depression, anxiety, and post-traumatic stress disorders).^10^ It is also a known risk factor leading to new onset dementia.^11^ Both conditions lead to decreased life satisfaction,^12^^,^^13^ and a significant burden on patients and caregivers.^14^

Prevention is considered the most effective way to control delirium,^15^ and more than two-thirds of delirium cases are preventable.^16^ Current detection methods rely on periodic assessments by nurses, such as the confusion assessment method (CAM). The CAM includes 4 components: (1) acute onset and fluctuating course, (2) inattention, (3) disorganized thinking, and (4) altered level of consciousness.^17–19^ However, CAM has the following limitations. First, it cannot continuously track patient status. A common interval for CAM assessments is every 12 h for hospitalized adults, which might lead to delays in delirium recognition and proactive interventions.^20^^,^^21^ Second, CAM can accurately determine the presence of delirium when it occurs, but cannot predict future states. Third, a CAM assessment requires patient participation, which interrupts sleep and is unattainable for patients who are under deep sedation. Lack of early detection remains a pressing issue that hinders healthcare providers from providing timely and effective interventions, for example, ABCDEF Bundle.^16^^,^^22^

Previous studies have attempted to apply machine learning methods to predict delirium or delirium-related diseases; however, several gaps remain when using prediction models in real clinical settings.^23^ First, previous studies have primarily used data from clinical trials to develop models, which have strict criteria for patient selection and data sets that are generally more complete and smaller than typical clinical use cases.^24^ For example, a recent model was developed on a dataset of 1026 patients with excluded dementia.^24^ Whereas epidemiological evidence suggests that the presence of dementia is a substantial contributor to delirium and can increase the risk of delirium by 2–5 times.^11^ Therefore, the model’s predictive performance in hospitalized patients is yet to be validated. Second, another previous study that used International Classification of Diseases (ICD) codes to label delirium yielded a presence rate of only 1.5%.^25^ However, it has been shown that using ICD only identifies 18% of the total delirium cases, so the model would result in a large number of delirious patients undetected.^26^ Third, the existing delirium prediction models were insufficient to account for temporal data. Most of them predicted delirium based on the features collected, with each record considered as an independent case. In clinical usage, each patient usually has multiple CAM assessments during hospitalization, and each assessment and the associated feature values should be considered as continuous data that may affect the subsequent delirium status.

The purpose of this study was to develop accurate deep learning models to predict new onset delirium in hospitalized adult patients. We proposed a method to utilize an LSTM-based model to capture temporal correlations to predict delirium status based on several previous CAM assessments and feature values in a time series. For patients without multiple CAM assessments yet, we used a machine learning model to predict delirium based on static data. Our study utilized a generalizable dataset that was routinely collected from Vanderbilt University Medical Center (VUMC)’s electronic health record (EHR) system for approximately 4 years. In addition, considering clinical practice, we predicted the new onset of delirium (ie, new positive CAM assessment)^27^ and provided visual interpretations of the predictions. The research was conducted at VUMC and was approved by the Vanderbilt University Institutional Review Board.

## MATERIALS AND METHODS

### Study design and population

We extracted all adult patients who had a CAM assessment between January 1, 2018 and October 1, 2021 in the intensive care unit (ICU) from VUMC’s clinical data warehouse. We excluded CAM assessments performed less than 12 h after the time of arrival on the unit and CAM assessments after new onset delirium. At VUMC, nurses conducted routine CAM assessments to assess delirium status in the ICU. The prediction label was based on the result of the CAM assessment (ie, positive or negative). Diagnosis of delirium using CAM requires the presence of feature 1 (acute onset or fluctuating course) and feature 2 (inattention) and either feature 3 (disorganized thinking) or feature 4 (altered level of consciousness).^27^

### Data collection and preprocessing

The goal of our study is to predict delirium before it occurs. We assessed 3 time windows: 6, 12, and 24 h before the onset of the delirium event. For each time window, we collected the latest values from model features generated at least that many hours before the next CAM assessment. For example, when the time window was 6 h, we only considered data at least 6 h prior to the CAM assessment. We collected 896 features from the following EHR data domains: medications, vital signs, laboratory values, active problems, historical problems, type of surgery, social history, procedures, and hospital admission. For each feature, we calculated the missing rate in the training dataset and removed features with missing rates >0.99. We used the Clinical Classifications Software to map diagnosis codes into categories.^28^ The preprocessing process consisted of 3 steps: (1) imputation of missing values, (2) scaling, and (3) encoding categorical features. Categorical features were reported as counts and percentages. Numerical features were reported as mean with standard deviation (SD) and median with interquartile range (IQR).

### Machine learning model development and evaluation

We split the dataset at the patient level into a training dataset (80%) and a testing dataset (20%). The testing dataset was used as a hold-out dataset for external validation. We used 5-fold cross validation on the training dataset to tune hyperparameters in models. After obtaining the optimal hyperparameters, we developed models using the training dataset, then performed 1000-round bootstrapping with the hold-out testing dataset to report the results. We developed logistic regression, random forest, support vector machine, and LightGBM^29^ models. Gradient boosting decision tree models have been applied to other clinical tasks with excellent performance compared to traditional machine learning algorithms.^30–32^ We predicted the risk of new onset delirium within 6, 12, and 24 h, respectively. We reported outcomes in *F*1, accuracy, area under the receiver operating characteristic curve (AUC), recall, and precision. To evaluate the overall performance, we plotted receiver-operating characteristic curves and precision–recall curves. The receiver-operating characteristic is the ratio of sensitivity to (1-specificity). Models with a larger AUC are considered to have better performance. On the other hand, the precision–recall curve illustrates the trade-off between recall and precision. Models with high performance tend to have a balance of high recall and precision, yielding large *F*1 values. Machine learning model development and evaluation were done using the following packages: numpy, pandas, matplotlib, sklearn, and lightgbm.

### Statistical analysis

To compare the characteristics between patients with and without delirium in the cohort, we performed Welch *t* tests for numerical features and Chi-square tests for categorical features. To compare the performance of different models, we conducted a Friedman test^33^ on *F*1 values with a follow-up Nemenyi test^34^ for pairwise comparisons.^35^*P<*.05 was considered to be statistically significant.

### Model explainability

We calculated Shapley additive explanations (SHAP) values^36^ for each feature and applied the SHAP framework to interpret each prediction on the hold-out set. SHAP values are intended to explain complex “black-box” machine learning models, for example, neural networks and gradient boosting tree-based models.^36^ The SHAP framework provides a unique solution with important properties (local accuracy, missingness, and consistency) based on additive feature attribution methods and game theory. It is calculated by comparing the predicting differences in all possible combinations containing and withholding each feature. It shows better consistency and accuracy with human intuition compared to previous approaches to model interpretation.

### Machine learning and LSTM combined model

**Figure 1.**
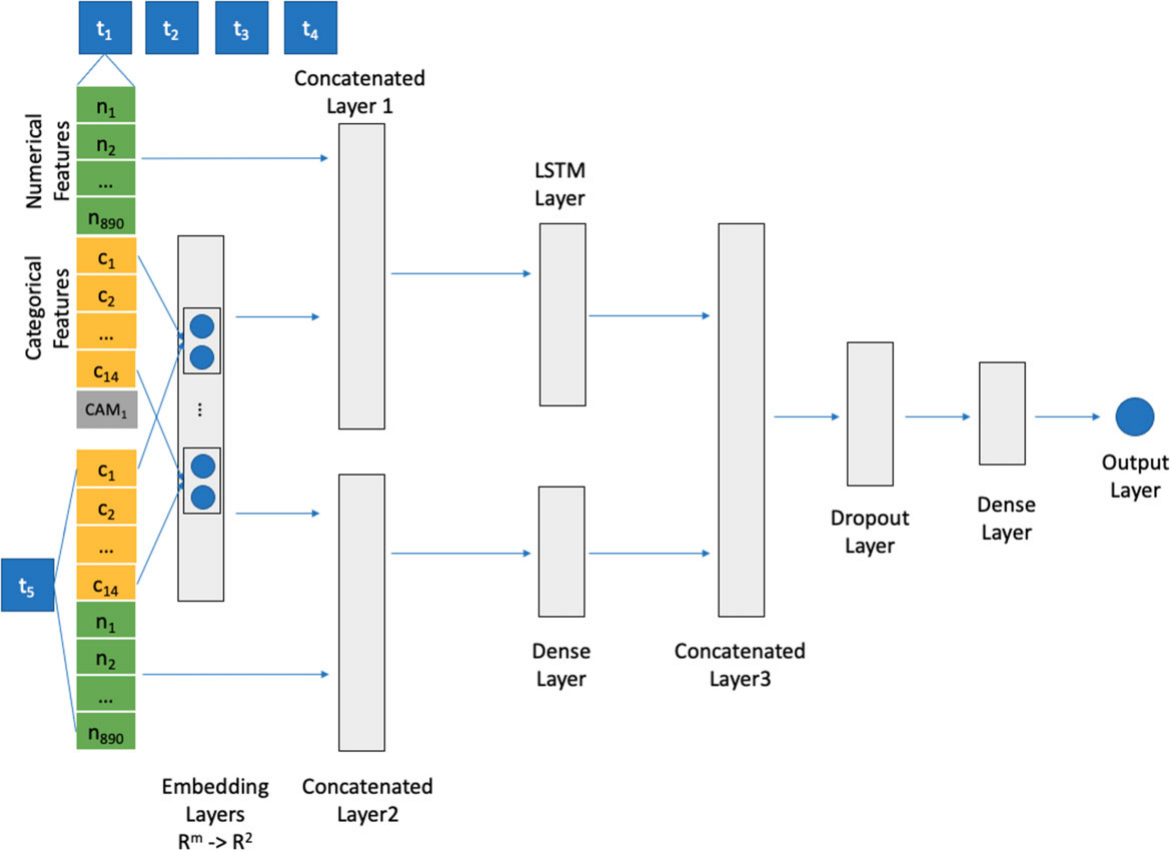
The proposed LSTM-based model. Abbreviation: LSTM: long short-term memory.

For data preprocessing, we used the same training and testing datasets as in the previous section on machine learning, with the data partitioned at the patient level such that our testing and training sets included nonoverlapping subsets of patients. The deep learning model includes 2 parts: (1) a fixed-length LSTM-based model and (2) a machine learning model. To develop the LSTM-based model, we selected encounters with at least 4 CAM assessments in the training set based on the median number of CAM assessments per hospitalization in our dataset of 4. To capture temporal relationships we chose the LSTM method, a state-of-the-art deep learning model designed to analyze sequential data.^37^ In the LSTM-based model, we developed embedding layers to convert each categorical feature into 2-dimensional dense real-valued vectors (*R*^2^). Numerical features were imputed using mean value, transformed using a standard scaler, and connected to the embedded vectors via a concatenated layer. In addition to the 4 previous CAM assessments and associated features, we developed a neural network to integrate into the LSTM model the most recent features generated at least 6 h prior to prediction through another concatenated layer. Our proposed LSTM-based model is shown in Figure 1. The units of the LSTM layer and the dense layer, the learning rate, and the dropout rate were tuned by using Hyperband Tuner in Keras, an efficient hyperparameter optimization approach widely used in deep learning.^38^ The model was trained using an Adam optimizer and a binary cross-entropy loss function. Second, for the delirium status in the first 4 assessments during each hospitalization in the testing dataset, we selected the optimal machine learning model developed in the previous section to make predictions.

## RESULTS

### Patient characteristics

A total of 331 489 CAM assessments from 34 035 patients with 39 567 encounters were included in the final dataset. The characteristics of patients are listed in Table 1. The median age of patients was 59 years with an IQR [44, 70]. A total of 37 246 were positive CAM assessments (11.2%). Patients in the delirium group were older, most had public insurance, and had longer length of stay (*P*<.001). Race and sex were not significantly different in 2 groups. We extracted 896 features: medications (195), vital signs (10), laboratory values (39), active problems (161), historical problems (84), type of surgery (108), social history (5), procedures (279), and hospital admission (13). Examples of features are presented in Table 2. The median number of CAM assessments in each hospitalization was 4 with an IQR.^2^^,^^9^

**Table 1.** Characteristics of patients

Characteristic	Delirium (*n* = 37 246)	Non-delirium (*n* = 294 243)	*P* value
Age (years)			
Mean (SD)	60.40 (16.95)	56.34 (17.60)	<.001
Median (IQR)	63 (50–73)	59 (44–70)	
Age groups (years), *n* (%)			<.001
18–29	617 (6.4%)	3225 (9.8%)	
30–39	686 (7.1%)	3390 (10.3%)	
40–49	1040 (10.8%)	4212 (12.8%)	
50–59	1763 (18.3%)	6179 (18.8%)	
60–69	2325 (24.1%)	7600 (23.1%)	
≥70	3209 (33.3%)	8290 (25.2%)	
Length of Stay			0.004
Mean (SD)	5.64 (8.05)	5.38 (6.70)	
Median (IQR)	3 (1–7)	3 (2–6)	
Race			0.079
White	7520 (78.6%)	25 266 (79.8%)	
Black/African American	1497 (15.7%)	4571 (14.4%)	
Asian	106 (1.1%)	359 (1.1%)	
American Indian	15 (0.2%)	67 (0.2%)	
Pacific Islander	9 (0.1%)	33 (0.1%)	
Unknown	417 (4.4%)	1381 (4.4%)	
Insurance type			<.001
Public	6568 (70.0%)	18 963 (60.9%)	
Private	2325 (24.8%)	10 097 (32.4%)	
Sex			
Male	5555 (59.2%)	18 254 (58.6%)	0.323
Female	3826 (40.8%)	12 877 (41.4%)	
Specialty (top 10)			<.001
Intensive care	1666 (26.1%)	4219 (21.7%)	
Neurological intensive care	896 (14.1%)	3818 (19.7%)	
Surgical intensive care	1084 (17%)	3696 (19%)	
Neurology	495 (7.8%)	1729 (8.9%)	
Burn surgery	294 (4.6%)	1279 (6.6%)	
Hematology and oncology	278 (4.4%)	1164 (6%)	
General internal medicine	472 (7.4%)	1099 (5.7%)	
Palliative care	747 (11.7%)	982 (5.1%)	
Cardiac intensive care	256 (4%)	436 (2.2%)	
Transplant	107 (1.7%)	412 (2.1%)	

**Table 2.** Examples of extracted features

Category	Number of features	Examples
Medications	195	Laxatives and cathartics, opioid analgesics, analgesic antipyretics non salicylate, antiemetic antivertigo agents, insulins, sodium saline preparations, heparin and related preparations
Vital signs	10	Height, weight, pulse, respiratory rate, systole blood pressure, diastole blood pressure, heart rate, MAP, BMI, SpO_2_
Laboratory values	39	Calcium, basophils, immature granulocytes, lactate whole blood, total hemoglobin whole blood, glucose whole blood, sodium
Active problems	161	Essential hypertension, fluid and electrolyte disorders, respiratory failure, diabetes mellitus without complication, cardiac dysrhythmias, deficiency and other anemia
History problems	84	Fluid and electrolyte disorders, respiratory failure, acute and unspecified renal failure, diabetes mellitus without complication, septicemia, diseases of white blood cells, cardiac dysrhythmias, essential hypertension
Surgery	108	Exploration laparotomy, prebuilt exploratory lap conversion, upper endoscopy, intraoperative nerve testing, transplant liver, cerebral angiogram
Social history	5	Race, ethnicity (Hispanic, not Hispanic), insurance (private/public), smoking status, education level
Procedure	279	XR AP chest portable, TYPE SCRN ABO RH AB SCRN, EKG electrocardiogram, update patient service team level of care, transfer patient, culture BACT BLD adult
Demographics and other information	13	Length of current hospital stay (days), age (years), sex, department, specialty, level of care, number of CAM assessments
Other measurements	2	RASS score, fall risk score

CAM: confusion assessment method; RASS: Richmond Agitation-Sedation Scale.

### Machine learning model performance

Machine learning model hyperparameters are listed in Supplementary Table S1. LightGBM outperformed other machine learning models on the hold-out testing dataset for predictions made 12 h before onset with an AUC score of 0.921 (95%CI: 0.918, 0.923) and an *F*1 score of 0.619 (95%CI: 0.611, 0.627). The random forest model had the lowest *F*1 score of 0.334 (95%CI: 0.323, 0.346). The neural network had the lowest AUC score 0.772 (95%CI: 0.767, 0.778). Other metrics are reported in Table 3. In the Friedman test, all metrics were significantly different across the 5 models (*P* <.001). In the Nemenyi post-hoc test, the LightGBM model had significantly higher *F*1 and AUC scores than the other models’ metrics (*P* =.001).

**Table 3.** Prediction results on the testing dataset (prediction time window = 12 h)

Model	Recall	NPV	Specificity	Precision	Accuracy	*F*1	AUC
Logistic Regression	0.759** **[0.754, 0.773]**	0.962 [0.961, 0.964]	0.834 [0.831, 0.836]	0.385 [0.379, 0.392]	0.825 [0.822, 0.828]	0.511 [0.506, 0.520]	0.879 [0.874, 0.883]
Support Vector Machine	0.372 [0.368, 0.386]	0.918 [0.917, 0.921]	0.969 [0.968, 0.971]	0.62 [0.620, 0.646]	0.897 [0.896, 0.901]	0.465 [0.464, 0.481]	0.88 [0.878, 0.889]
Random Forest	0.211 [0.203, 0.221]	0.902 [0.899, 0.904]	0.993** [**0.992, 0.993**]	0.797** [**0.780, 0.812**]	**0.898** [**0.896, 0.901**]	0.334 [0.323, 0.346]	0.908 [0.902 0.91]
Neural Network	0.405 [0.396, 0.416]	0.918 [0.916, 0.921]	0.914 [0.911, 0.916]	0.392 [0.382, 0.402]	0.853 [0.850, 0.856]	0.399 [0.390, 0.408]	0.772 [0.767, 0.778]
LightGBM	0.752 [0.742, 0.761]	0.964* [**0.962, 0.965**]	0.907 [0.905, 0.909]	0.526 [0.517, 0.535]	0.888 [0.886, 0.891]	0.619** [**0.611, 0.627**]	0.921** [**0.918, 0.923**]

The best result on each metric is shown in bold.

* *P* =.015; **P =.001.

Using the LightGBM model, we tested its predictive ability in different time windows: 6, 12, and 24 h. The LightGBM model had the best performance at 6 h before onset. The *F*1 score and AUC score were 0.626 [0.618, 0.634] and 0.927 [0.924, 0.929], respectively. It can accurately predict 75% of new onset delirium. Other performance metrics for the testing dataset with different time windows are presented in Table 4. In the Friedman test, all metrics identified significant differences between the LightGBM models for 3 different time windows. In the Nemenyi post-hoc test, the LightGBM 6 h model had significantly higher specificity, precision, accuracy, *F*1, and AUC than associated metrics for the other 2 models (*P* =.001). The recall and NPV of the LightGBM 6 h model were not significantly different compared to the LightGBM 12 h model, but were significantly higher than the metrics of the LightGBM 24 h model.

**Table 4.** Prediction results on the testing dataset using different time windows in the LightGBM models

	Recall	NPV	Specificity	Precision	Accuracy	*F*1	AUC
6 h	0.75 [0.740, 0.759]	**0.964** [**0.962, 0.965**]	0.911** [**0.909, 0.914**]	0.537** [**0.527, 0.546**]	0.892** [**0.889, 0.894**]	0.626** [**0.618, 0.634**]	0.927** [**0.924, 0.929**]
12 h	**0.752** [**0.742, 0.761**]	**0.964** [**0.962, 0.965**]	0.907 [0.905, 0.909]	0.526 [0.517, 0.535]	0.888 [0.886, 0.891]	0.619 [0.611, 0.627]	0.921 [0.918, 0.923]
24 h	0.694 [0.684, 0.704]	0.955 [0.954, 0.957]	0.897 [0.895, 0.899]	0.48 [0.471, 0.489]	0.872 [0.870, 0.875]	0.568 [0.560, 0.576]	0.893 [0.890, 0.897]

AUC: area under the receiver operating characteristic curve.

** *P* =.001 (assess if the LightGBM 6 h model outperform the LightGBM 12 h model and LightGBM 24 h model).

**Figure 2.**
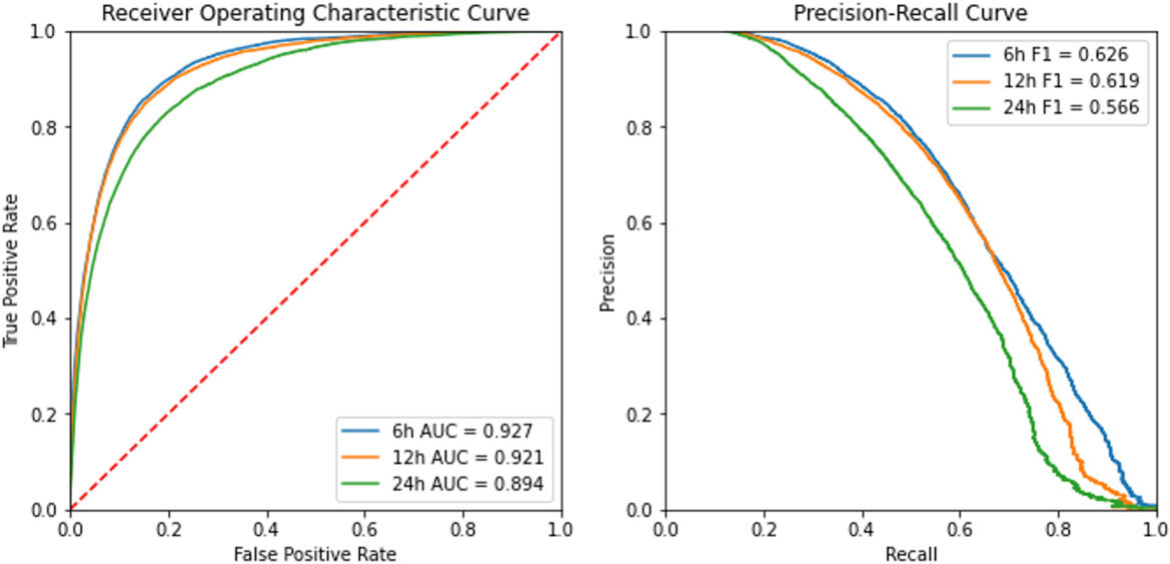
Performance of LightGBM model to predict new onset of delirium within 6, 12, 24 h. (Left) Receiver-operating characteristic curves. (Right) precision–recall curves.

The AUCs and the precision–recall curves are shown in Figure 2. The precision–recall curves of the LightGBM 6 h model and the LightGBM 12 h model were close; however, when the recall was fixed in a large value, the precision value of the LightGBM 12 h models was much smaller than the precision value of the LightGBM 6 h model. This suggests that the LightGBM model has a more robust performance in predicting new onset delirium within 6 h.

### Model explainability

**Figure 3.**
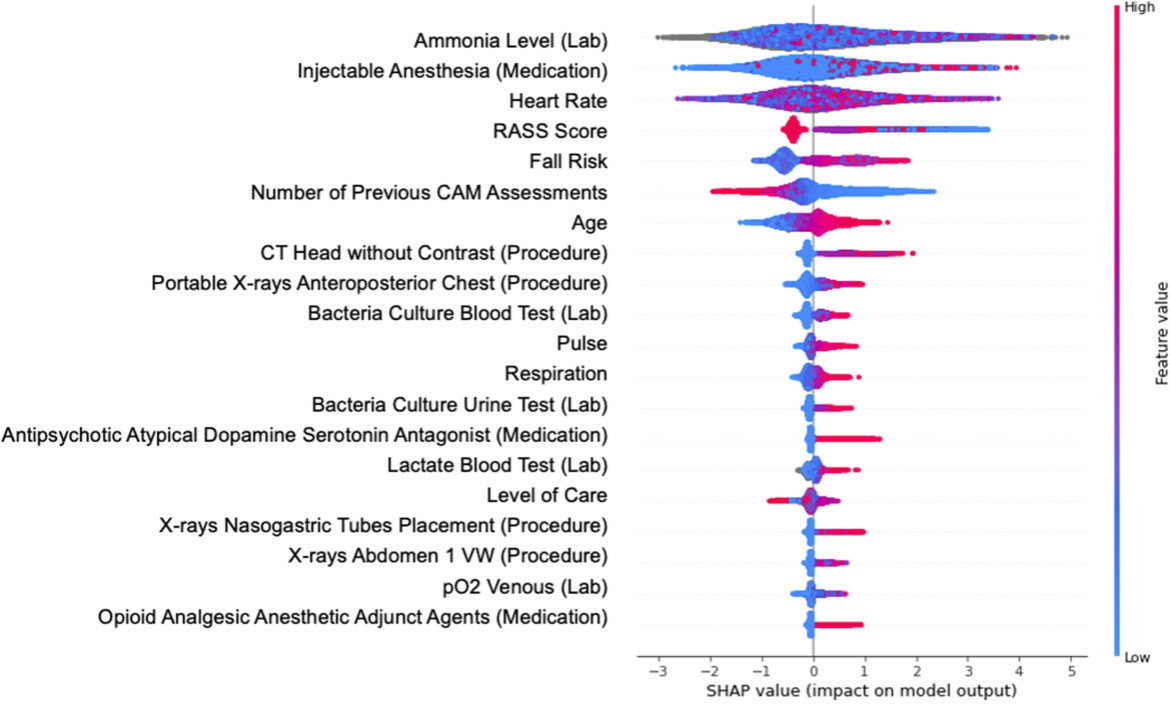
The SHAP summary plot. Level of care: for example, stepdown, general surgery, general medicine, and ICU. SHAP: Shapley additive explanations.

Using the mean absolute SHAP values, we determined the top 20 features including age, heart rate, Richmond Agitation-Sedation Scale (RASS) score, fall risk, pulse, respiratory rate, level of care, and the number of previous CAM assessments in this encounter. We also identified 3 laboratory values (ammonia level, lactate blood test, and pO2 venous), 3 medications (intravenous anesthesia, atypical antipsychotics, and opioid analgesic anesthetic adjunct agents), and 6 procedures (eg, CT head without contrast and portable X-rays anteroposterior chest) as important features for predicting new onset delirium. In Figure 3, we presented the relationships between their values and the effect of the model output.

**Figure 4.**
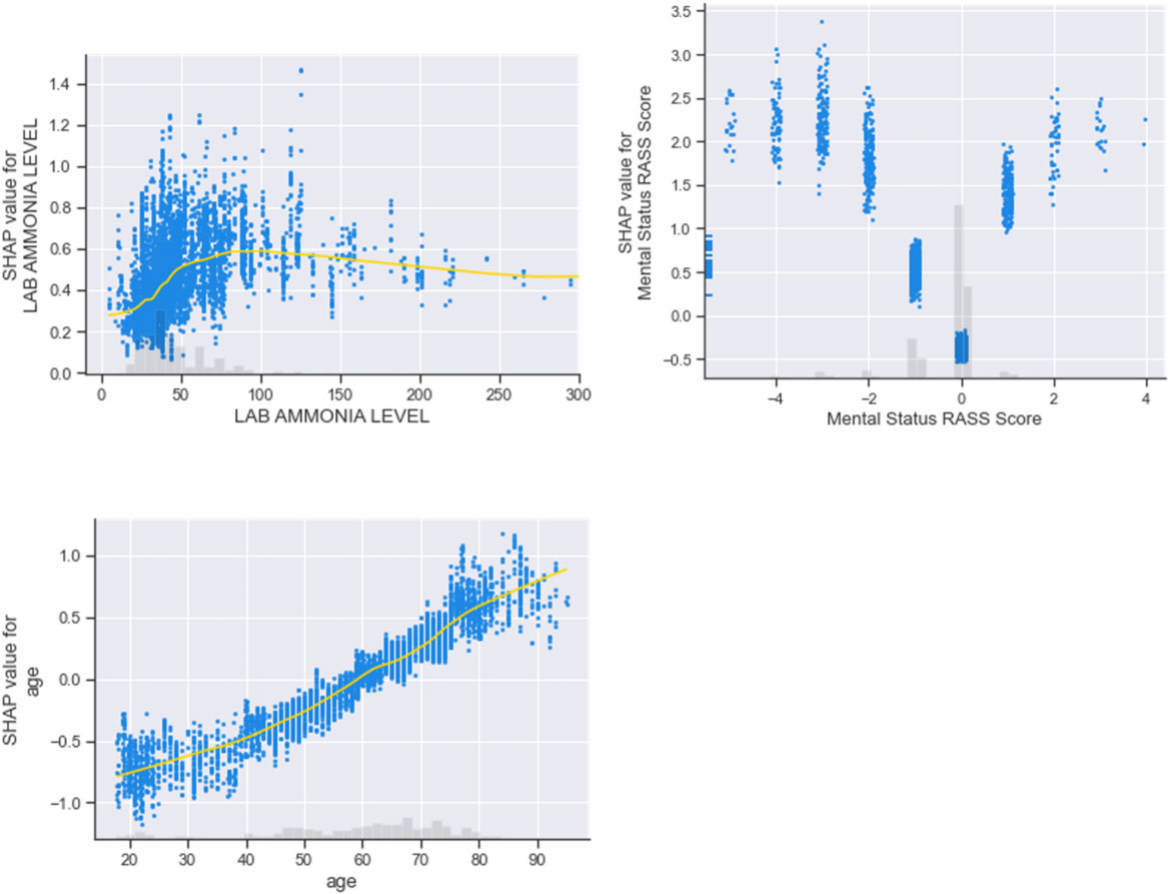
Scatter plots of the relationships between features (ammonia level [Lab], RASS score, age) and SHAP values. RASS: Richmond Agitation-Sedation Scale; SHAP: Shapley additive explanations.

Furthermore, we provided scatter plots (Figure 4) for several important identified features (eg, ammonia level, RASS score, and age). The gray histogram shows the distribution of values. For continuous features, we also added yellow lines to represent regression lines. For example, when ammonia levels exceeded the normal range (15–45 µ/dL), SHAP values increased and the effect on the model results became larger. For the RASS score, the effect on the prediction model was minimal when the patient was alert and calm (RASS score = 0). In addition, negative RASS scores had a greater impact on model prediction compared to positive RASS scores. For the age, we observed that the SHAP value increased when the patient’s age increased.

**Figure 5.**
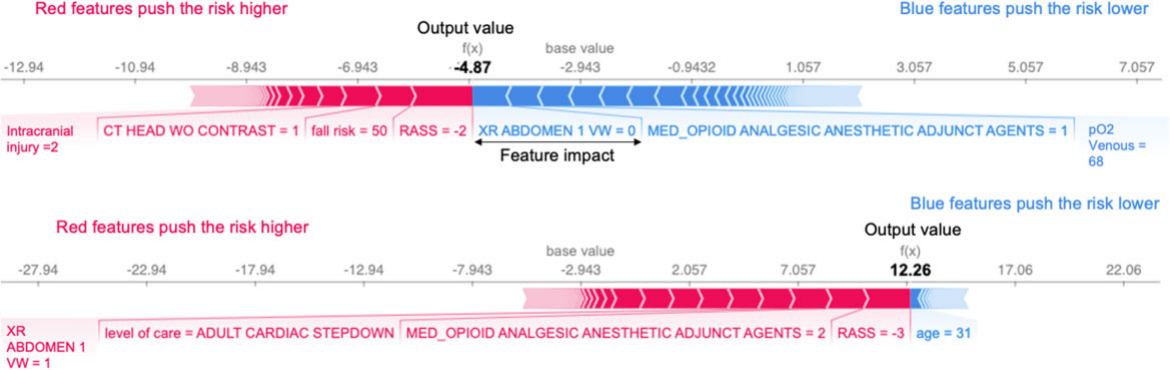
Examples of negative (top) and positive predictions (bottom) for new onset delirium.

In addition to the overall effect, we applied the SHAP framework to explain individual cases by providing influential features. Figure 5 shows 2 examples—a negative prediction (top) and a positive prediction (bottom). Features in blue represent features that contribute to a lower risk while features in red will push up the risk. These visualizations give users detailed information about how the model makes predictions and allow them to make appropriate interventions before the new onset delirium.

### Machine learning and LSTM combined model performance

The tuned LightGBM model was selected to predict the delirium status for first 4 assessments. The LSTM-based model with an AUC score of 0.966 [0.963, 0.969] and *F*1 score of 0.883 [0.878, 0.889] was used to predict delirium based on at least 4 assessments. Model hyperparameters are listed in Supplementary Table S1. Because we wanted to be able to make predictions for patients with fewer than 4 prior CAM assessments, we created a final model (LightGBM+LSTM) which used the LightGBM model for predictions where there were fewer than 4 prior CAM scores, and then switched to the more accurate LSTM once at least 4 scores had been recorded. The combined LightGBM+LSTM model had an AUC score of 0.952 [0.950, 0.955] and an *F*1 score of 0.759 [0.755, 0.765]. Other metrics are reported in Table 5. In the Friedman test, all metrics from the combined model were significantly different from the original LightGBM model (*P* <.001). In the Nemenyi post-hoc test, the *F*1 and AUC scores were significantly higher for the combined model (*P* =.001).

**Table 5.** Prediction results on the testing dataset using different time series models (LSTM: long short-term memory)

	Recall	NPV	Specificity	Precision	Accuracy	*F*1	AUC
LightGBM (baseline)	0.75 [0.740, 0.759]	0.964 [0.962, 0.965]	0.911 [0.909, 0.914]	0.537 [0.527, 0.546]	0.892 [0.889, 0.894]	0.626 [0.618, 0.634]	0.927 [0.924, 0.929]
LSTM-based model	0.858 [0.850, 0.864]	0.980 [0.979, 0.981]	0.988 [0.987, 0.989]	0.911 [0.905, 0.919]	0.972 [0.970, 0.973]	0.883 [0.878, 0.889]	0.966 [0.963, 0.969]
LightGBM+LSTM	0.823 [0.817, 0.827]	0.975 [0.974, 0.976]	0.953 [0.951, 0.954]	0.704 [0.699, 0.712]	0.937 [0.935, 0.939]	0.759** [0.755, 0.765]	0.952**[0.950, 0.955]

AUC: area under the receiver operating characteristic curve.

The best result on each metric is shown in bold.

** *P* =.001 (assess if the combined model outperforms the LightGBM).

**Figure 6.**
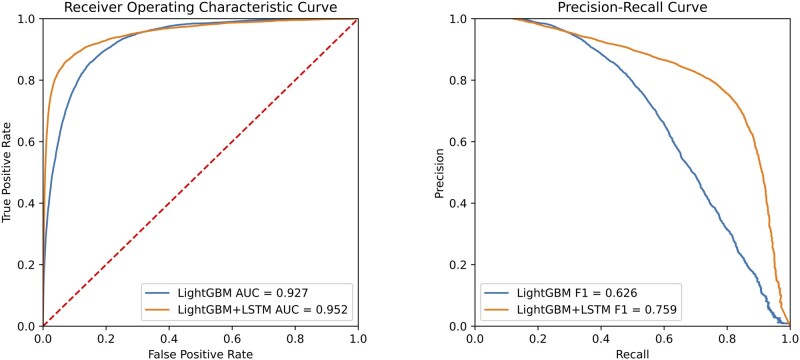
Performance of the LightGBM model and the combined model (LightGBM+LSTM) to predict the new onset delirium within 6 h. (Left) Receiver-operating characteristic curves. (Right) Precision–recall curves. LSTM: long short-term memory.

The AUCs and the precision–recall curves are shown in Figure 6. The AUCs of the LightGBM model and the combined model were similar; however, the precision–recall curves were different. When the recall was set to 0.8, the precision value of the combined model increased from 0.497 to 0.751 compared to the LightGBM model, an increase of 51%. The increments in precision values for other fixed recall values are reported in Table 6.

**Table 6.** Increments of precision values in different fixed recall values (LSTM: long short-term memory)

Fixed recall	Precision		Increment (%)
	LightGBM	LightGBM+LSTM	
0.90	0.378	0.499	32%
0.85	0.449	0.644	43%
0.80	0.497	0.751	51%
0.75	0.537	0.807	50%
0.70	0.572	0.844	48%

## DISCUSSION

### Principal findings

In this study, we developed a novel LSTM and LightGBM combined model to predict new onset delirium and evaluated the predictive capability of the model using EHR data generated directly from routine healthcare activities. This algorithm has better performance than the traditional machine learning model. It has the potential to be implemented as a clinical decision support (CDS) tool integrated into an EHR system. This means healthcare providers can obtain high performance risk assessments between manual CAM assessments and be able to provide proactive and timely interventions for high-risk patients.

The important features found in the LightGBM model are supported by clinical evidence. For example, a systematic review reported that the elevated levels of ammonia were associated with severe hepatic encephalopathy,^39^ a cause of delirium.^40^ The 3 medications found (injectable anesthesia, antipsychotic atypical dopamine serotonin antagonist, and opioid analgesic anesthetic adjunct agents) are also mentioned in other studies.^1^^,^^41^ Previous evidence suggests that older patients are at higher risk of delirium when exposed to anesthetics^1^ and higher risk of postoperative delirium when exposed to psychoactive drugs (benzodiazepines, opioids).^41^ Respiration rates and RASS scores have also been identified as significant predictors in previous prediction models.^24^ Although imaging is part of the predictive model, we are aware that imaging decisions may be based on clinical suspicion and/or protocolized care (eg, chest X-ray to evaluate endotracheal tube position). Therefore, the interpretation of imaging data would require clinical correlation. In the model explanation, we observed that the negative RASS scores had a greater impact on model predictions than positive RASS scores, suggesting that our model appears to be more capable of predicting patients with hypoactive delirium. Hypoactive delirium is an important subtype of delirium that is usually more common than hyperactive delirium.^42–44^ In addition, long durations of hypoactive delirium lead to long-term cognitive decline.^45^ However, because the patient exhibits fewer behavioral problems,^46^^,^^47^ it is often difficult to detect resulting in underreporting.^44^ In this study, we also developed other Bidirectional Encoder Representations from Transformers (BERT)-based models for analyzing clinical notes and found that neither clinical notes alone nor in combination with unstructured data could achieve higher performance in predicting new onset delirium.

We found that using an LSTM-based model to treat historical CAM assessments and associated features as longitudinal data can substantially improve predictive performance. It indicates that the trajectory of historical data may also be informative in predicting delirium. This finding is consistent with other disease predictions, for example, heart disease.^48^ In addition, we combined LSTM with machine learning to provide predictions at the beginning of the time series, which was often ignored by previous time series studies of healthcare data. We also found that using the time interval of CAM assessments as the interval of the timestamps to integrate features is feasible in providing accurate predictions. Specifically, for each time point in our time series corresponding to a CAM assessment, features were selected from data generated 6 h prior to that CAM assessment. Previous studies typically aggregate data on an hourly basis, potentially generating more noise and imposing higher demands on model training. We identified a recent study that developed an LSTM-based model to predict delirium status at least 24 h after hospitalization based on 21 features.^49^ Our study used a more extensive set of over 900 features, while the machine learning part we introduced in the combined model could provide predictions when there was not enough historical data to run the LSTM-based model. In addition, as a critical step for implementation in the clinic, the performance of the prediction model should be considered. In the reported model, the maximum AUC was 88.39% with a precision and recall of 37.52% and 86.18%, respectively, that is, only 38 out of 100 predicted delirium diagnoses will occur, which would place an additional burden on health providers, especially in the ICU environment.

### Limitations

This study has several limitations. First, we developed models based on a dataset from a single medical center. Exploring the predictability of this model on other healthcare systems might add more value. However, it should be noted that the dataset was extracted from a large tertiary referral center with a broad catchment area. In addition, we used a hold-out testing dataset containing different patients for external validation. Third, as a retrospective study, the impact of predicting new onset delirium on patient outcomes is still unknown.

### Future work

Future work in this area should link delirium prediction with evidence-based actions through clinical decision support formats. It includes designing interactive interfaces, exploring better presentations to explain model behavior based on clinician needs, implementing it in the workflow, and further exploring the impact of the model on clinician behavior as well as patient outcomes. Another direction is to predict different types of delirium (ie, hypoactive delirium, hyperactive delirium, and mixed delirium) and to provide clinicians with corresponding actionable interventions for each type through CDS tools.

## CONCLUSION

Delirium remains a serious risk factor for older patients in the ICU and is one of the key directions for aging research. Early detection of new onset delirium in the clinical workflow is a critical step to enhancing patient monitoring and improving patient outcomes. We developed a deep learning prediction model for new onset delirium within 6 h using data generated directly from the EHR. The LSTM layer inside the model could capture the temporal relationships in historical data. This new model has excellent performance in predicting new onset delirium, which provides a solid technical basis for the intelligent CDS tool for delirium prediction in a future implementation study.

## FUNDING

This work was supported by NIH grant: R01AG062499-01 and K99LM014097-01.

## AUTHOR CONTRIBUTIONS

SL conducted feature identification, data extraction, model developing, statistical analysis, and drafting the work. SL, AM, JS, AW, BS, TR, TK, and ER helped to design experiments and revise the drafted manuscript. SL and TK performed a literature review. All authors approved the submitted version.

## SUPPLEMENTARY MATERIAL

Supplementary material is available at *Journal of the American Medical Informatics Association* online.

## CONFLICT OF INTEREST STATEMENT

None declared.

## Supplementary Material

ocac210_Supplementary_Data

## Data Availability

The data underlying this article cannot be shared publicly due to patient healthcare data privacy protection requirements.
