# Supplementary material for: New onset delirium prediction using machine learning and long short-term memory (LSTM) in electronic health record
Source: J Am Med Inform Assoc. 2022 Oct 27;30(1):120–31. doi: 10.1093/jamia/ocac210 (PMC9748586; doi:10.1093/jamia/ocac210)
Supplement: ocac210_Supplementary_Data [file ocac210_supplementary_data.pdf]

Supplementary Material

Table 1. Hyperparameters used in machine learning models.

| Model | Hyperparameter |
|---|---|
| Logistic regression | solver='sag', C=0.01 |
| Random forest | max_features= 'sqrt', max_depth=50, n_estimators=2500 |
| Support vector machine | max_iter=1000, learning_rate='optimal', eta0=1e-8, alpha= 0.01 |
| Neural network | hidden_layer_sizes=(256,), early_stopping=True,alpha=1e-5, batch_size=500, learning_rate_init= 0.001,n_iter_no_change=3 |
| LightGBM 6h | Objective='binary', boosting_type='gbdt', num_leaves=246, feature_fraction=0.978, bagging_fraction=0.870, bagging_freq=4, min_child_samples=82, lambda_l1=1.323e-05, lambda_l2=2.003e-05 |
| LightGBM 12h | Objective='binary', boosting_type='gbdt', num_leaves=255, feature_fraction=0.0.680, bagging_fraction=1.0, bagging_freq=0, min_child_samples=10, lambda_l1=0.0005, lambda_l2=0.003 |
| LightGBM 24h | Objective='binary', boosting_type='gbdt', num_leaves=256, feature_fraction=0.874, bagging_fraction=0.788, bagging_freq=1, min_child_samples=43, lambda_l1=0.00032, lambda_l2=0.709 |
| LSTM+LightGBM | LSTM_units = 352, NN_units=128, dropout = 0.5, learning_rate=0.0001, Objective='binary', boosting_type='gbdt', num_leaves=246, feature_fraction=0.978, bagging_fraction=0.870, bagging_freq=4, min_child_samples=82, lambda_l1=1.323e-05, lambda_l2=2.003e-05. |
